# Gene expression array profile of human osteosarcoma

**DOI:** 10.1038/sj.bjc.6601389

**Published:** 2003-12-09

**Authors:** P Leonard, T Sharp, S Henderson, D Hewitt, J Pringle, A Sandison, A Goodship, J Whelan, C Boshoff

**Affiliations:** 1 Wolfson Institute for Biomedical Research, University College London, Gower Street, London, UK; 2 Institute of Orthopaedics and Musculo-Skeletal Science, University College London, London, UK; 3 Royal National Orthopaedic Hospital NHS Trust, Brockley Hill, Stanmore, UK; 4 University College London Hospitals NHS Trust, Mortimer Street, London, UK

**Keywords:** osteosarcoma, gene expression, microarray

## Abstract

The aims of this pilot study were to determine whether needle and open biopsies from osteosarcoma (OS) provide sufficient quality of mRNA for cDNA array analyses to gain insights into the expression profile of OS. A total of 22 samples collected from OS were used for array analyses. A primary cell culture was also established from one of the OS biopsies. Total RNA was extracted and probes were generated for cDNA arrays. cDNA probes were made for all the 22 samples. Two of these samples were needle core bone biopsies. Statistical analysis confirmed the reliability of array data obtained in 16 of the 22 samples. Known genes involved in bone metabolism and osteosarcoma were identified as highly expressed, and the putative new marker Ezrin was also identified. Confirmatory immunohistochemical staining using the Ezrin antibody was performed in a selection of samples.

Osteosarcoma (OS) is the commonest high-grade primary bone malignancy. It characteristically affects the long bones of teenagers with a peak age incidence between 13 and 16 years, but may also occur with a lower incidence throughout adulthood. However, it remains a rare tumour and only 130 and 1000 new cases are diagnosed each year in the UK and US, respectively (Dorfman & Czerniak, 1995; Whelan and Cannon, 2002).

Osteosarcoma may be suspected on the basis of characteristic clinical and radiological features. The diagnosis is generally established by core needle biopsy after which preoperative combination chemotherapy is followed by surgery, either with limb salvage or less commonly nowadays by amputation. Chemotherapy is continued postoperatively for up to 6 months.

Before the introduction of cytotoxic treatment, less than 20% of patients with OS were cured (Stiller and Bunch, 1990). Although adjuvant and neoadjuvant cytotoxic treatment and more refined surgical procedures have increased 5-year survival to over 60%, treatment remains crude, invasive and highly toxic for the majority of patients (Ferguson & Goorin, 2001).

Since the 1970s, histological response to presurgical chemotherapy has provided the most consistent and reliable prognostic indicator: those patients with localised disease whose tumours have undergone more than 90% necrosis have a 5-year survival in the region of 70%, while for those in whom the response falls short of 90%, survival rarely exceeds 40–50% (Davis *et al*, 1994). Alteration of chemotherapy postoperatively for those patients with a poor response has as yet not been demonstrated to improve outcome, and the relative rarity of OS has stifled the development and testing of novel agents for this disease, resulting in little progress to improve survival within the last decade.

Currently, most tumours are categorised on the basis of morphology. The identification of markers that distinguish subtypes of tumours and which may have prognostic and therapeutic implications is urgently needed. Gene expression microarrays (GEM) with bioinformatics analysis can be used to identify the molecular fingerprint (or signature) of an individual patient's tumour, and simple hierarchical clustering has already led to the identification of new classes of cancer that transcend the distinctions based on morphology and immunohistochemistry (DeRisi *et al*, 1996; Perou *et al*, 1999; Alizadeh *et al*, 2000; Perou *et al*, 2000). More advanced computational methodologies including supervised learning (Shipp *et al*, 2002) and artificial neural networks have also been used to define subtypes of breast (Seker *et al*, 2002) and colorectal cancer (Selaru *et al*, 2002).

Osteosarcoma may be particularly amenable to GEM analysis, as samples are routinely obtained before and after chemotherapy. The gene expression profile prior to therapy may thus be correlated with histological response after neoadjuvant treatment. A potential drawback, however, is that the small core biopsy samples obtained for diagnostic purposes may contain a proportion of calcified bone and therefore be unsuitable for the generation of cDNA probes for array hybridisation technologies.

The aims of this study were to determine if sufficient high-quality mRNA could be obtained from both needle core and open biopsy OS samples from which to generate cDNA probes to obtain array data.

## MATERIALS AND METHODS

### Patient and clinical tissue

In total, 22 fresh frozen high-grade OS specimens were obtained from surplus material used for diagnostic purposes. Clinical characteristics of 16 patients included in the analysis are summarised in (Table 1

**Table 1:** Patient details

**Id number**	**Age**	**Gender**	**Histological subtype**	**Type of sample % necrosis**	**Time elapsed since cytotoxic chemotherapy**
Loc-131	7	F	Osteoblastic	Diagnostic needle core biopsy	4 months
Pre-147	12	F	Mixed differentiation	Diagnostic needle core biopsy	None
Pre-283	14	M	Common	Resection sample	None
Pre-124	71	F	Osteoblastic	Resection sample	None
Loc-181	14	F	Common	Resection sample	None
Post-720	8	M	Common	Post chemotherapy resection < 90% necrosis	2–3 weeks
Post-125	16	F	Fibroblastic	Post chemotherapy resection < 90% necrosis	2–3 weeks
Post-876	22	M	Common	Post chemotherapy resection 70% necrosis	2–3 weeks
Post-139	16	F	Osteoblastic	Post chemotherapy resection < 90% necrosis	2–3 weeks
Post-397	18	M	Osteoblastic	Post chemotherapy resection < 90% necrosis	2–3 weeks
Post-307	23	M	Common	Post chemotherapy resection 50% necrosis	2–3 weeks
Post-111	13	F	Osteoblastic	Post chemotherapy resection < 90% necrosis	2–3 weeks
met-848	14	M	Telangiectatic	Metastatectomy	2–3 weeks
met-040	12	F	Osteoblastic	Metastatectomy	2–3 weeks
met-576	16	F	Common	Metastatectomy	14 months
met-c738	13	M	Osteoblastic	Metastatectomy	23 months
met-738	13	M	Osteoblastic	Metastatectomy	23 months

). Ethical Committee approval was obtained from The Royal National Orthopaedic Hospital NHS Trust for this study.

### Cell culture

From one of the samples (met-738), primary cells were cultured for array analysis (met-c738). The sample was placed in 165iu collagenase (Collagenase type 2 GIBCO™ Invitrogen Corporation, Daisley, UK) to disaggregate the cells. Primary cell culture growth was maintained in DMEM with 10% FCS. To confirm that the cells were from osteoblastic lineage and so represented OS cultured from the tumour sample, electron microscopy and alkaline phophatase staining were performed. Osteocalcin levels were measured in the supernatant of the flasks in which the cells were cultured.

### Histopathology

Osteosarcoma biopsies first underwent imprint staining for alkaline phosphatase to rapidly confirm the diagnosis before freezing for RNA preservation. Adjacent samples were histologically confirmed by Haematoxylin and Eosin staining. The extent of necrosis within the postchemotherapy samples varied between 50 and 70%.

### cDNA arrays

All experiments were performed on Human Genefilters™ GF211 (〈http://www.resgen.com〉) nylon microarrays with 4324 known human cDNA probes selected from Unigene. Osteosarcoma tissue was dissected into approximately 0.5 cm^3^ pieces. Each piece was placed into a sterile cryotube, immediately snap-frozen and stored in liquid nitrogen until ready for RNA extraction. For RNA extraction, frozen tumour tissue was placed in a guanidine–thiocynate-containing buffer, RLT as supplied by RNeasy mini kit (Qiagen® Ltd, West Sussex, UK). Additional *β*-mercaptoethanol (*β*-ME) was added as per the manufacturer's instructions. The specimen was initially homogenised, using a rotary star homogeniser then underwent further homogenisation using a Q1A shredder (Qiagen® Ltd, West Sussex UK). RNA extraction was then followed as per the manufacturer's protocol using an RNeasy mini kit (Qiagen® Ltd). For RNA extraction from cell lines and primary cell cultures, the denaturing buffer was added directly to the sterile tube containing the cell pellet. The sample was homogenised using a Q1A shredder (Qiagen® Ltd). RNA extraction followed as per the manufacturer's protocol using an RNeasy mini kit (Qiagen® Ltd). Integrity of total RNA was confirmed by the presence of 28S and 18S ribosomal bands on 1% agarose gels. The quantification and purity was assessed by spectrophotometry. Single-stranded cDNA was synthesised as per Research Genetics protocol (〈http://www.resgen.com〉). Total RNA (5 *μ*g) was used to make each single probe. Duplicate probes were made from one pool of RNA from each individual patient. The probe was radiolabelled with ^33^PdCTP (ICN Radiochemicals, Amersham, UK), then purified from unincorporated nucleotides using a Biospin 6 column (Bio-Rad, Hertfordshine, UK #732–6002). The radiolabelled probe was hybridised overnight to a prehybridised nylon filter. Prehybridisation in a solution of MicroHyb (Research Genetics, Huntsville, AL, USA) to which Human Cot-1 (Human Cot-1 DNA, Life technologies now Invitrogen Corporation, Daisley, UK # HYB125.GF) and poly dA (Research Genetics Cat. #POLYA.GF, 1 *μ*g *μ*l^−1^, Huntsville, AL, USA) were added was performed for at least 2 h. Following hybridisation with the radiolabelled probe, the filters were washed using 0.5–1% concentrations of sodium dodecyl sulphate (SDS) and 2X–0.5X SSC. The filters were exposed to a phosphor screen for 48 h and then scanned using a Cyclone™ Packard Instrument Company, Meridan, USA.

### Data analysis

Image analysis was performed using Research Genetics *Pathways*™ software. The expression values were then log scaled (base-2) and the arrays median scaled to the array with the median overall intensity. The quality of each array hybridisation was assessed by a set of criteria including a s.d. and skew of the logged gene expression values between 1–2 and 2–3 respectively. Nontypical hybridisations were excluded from subsequent analysis. We used duplicates for all arrays that had a correlation coefficient of 0.8 or greater. Data generated from met-576, post-307, post-397 and loc-R1 were not duplicate samples but met the remaining quality control criteria. Average linkage hierarchical clustering of the log transformed ratio values was performed using the program Cluster™ after filtering the genes to remove nonvarying data (SD< 0.5 of log transformed values) and presented using Treeview™ (http:www.microarrays.org/softw are).

### Immunohistochemistry

Immunohistochemistry (IHC) for Ezrin was achieved according to the standard streptavidin–biotin-labelling method. Briefly, antigen retrieval was performed by treatment in a microwave for 25 min in high-pH antigen retrieval solution (DAKO, Cambridgeshire UK). A rabbit polyclonal antibody (1 : 500 From ICR, London, UK (Dr Richard Lamb) and an anti-rabbit ChemMate Detection Kit (DAKO) were used as per the manufacturer's instructions for detection of the Ezrin protein. Two samples used corresponded to the RNA samples used in the RT–PCR confirmatory study (post-131 and met-738). Five other samples represent tissue used in the microarray data set (pre-147, pre-283, post-125, post-139 and met-740).

## RESULTS AND DISCUSSION

### 

Problems related to acquiring sufficient mRNA for meaningful array analyses were exemplified in a recent study attempting such analysis on fine needle aspiration (FNA) samples of breast cancer tissue. Only 15% of FNA samples were suitable for array analysis (Assersohn *et al*, 2002). Although the biopsies used in our study represent more tissue than is present in FNA samples, the fact that calcified bone tissue is part of these biopsies could potentially have decreased the ability to extract sufficient quality RNA. In the present study, all 22 tumour samples provided sufficient quality total RNA for making radiolabelled cDNA probes. Subsequent array data were derived from 16 of the 22 samples. The array data from the outstanding six samples were excluded from further analysis as they did not meet the statistical quality control criteria (see Materials and Methods). The patient details of the remaining 16 samples are detailed in Table 1.

The most strongly expressed genes within the patient tumour samples (above the 99th percentile of mean expression) contained many known bone markers (see Table 2

**Table 2:** Genes among the 99th most highly expressed on average in all patient samples showed a distinct bone-like profile

**Accession**	**Gene name**
AA076063	Caldesmon
AA397824	Dopachrome tautomerase
AA521431	Profilin
AA155913	Matrix Gla protein
AA872001	Annexin VI
AA490172	Collagen-I,*α*-2
AA411440	Ezrin^a^

**Figure 1:**
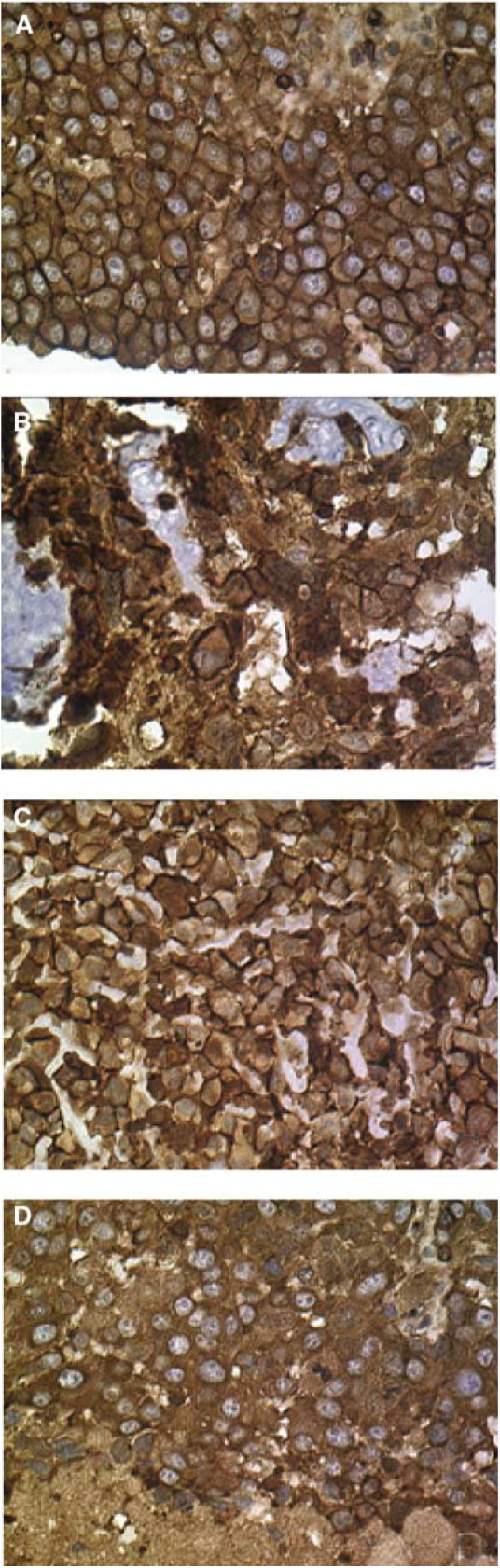
Immunohistochemical staining for Ezrin. **(A**) Prechemotherapy diagnostic biopsy sample. (**B**) Postchemotherapy resection sample. (**C**) Local recurrence diagnostic biopsy. (**D**) Metastatic sample. All pictures × 40.

These genes were consistently high in allpatients.

a Excepting Ezrin that lay just below the 99th percentile of expression.

). In particular, Collagen-1,alpha-2 and Matrix GLA are the major constituents of bone matrix. Both of these were high in all samples demonstrating the high osteoid content of all patient samples. Another highly expressed gene Ezrin is reported for the first time in primary OS samples (just below the 99th percentile). This gene is of particular interest due to its previously reported association with metastasis of an OS cell line in a murine model (Khanna *et al*, 2001). Interestingly, while we found no marked difference in gene expression between the different samples (see below) histopathological staining showed a distinctive membranous pattern in the metastatic samples (Figure 1D

), while primary and local recurrences showed both membrane and cytosolic staining (see Figure 1A–C). A similar finding was previously reported in uterine endometrioid adenocarcinoma (Ohtani *et al*, 1999).

**Figure 2:**
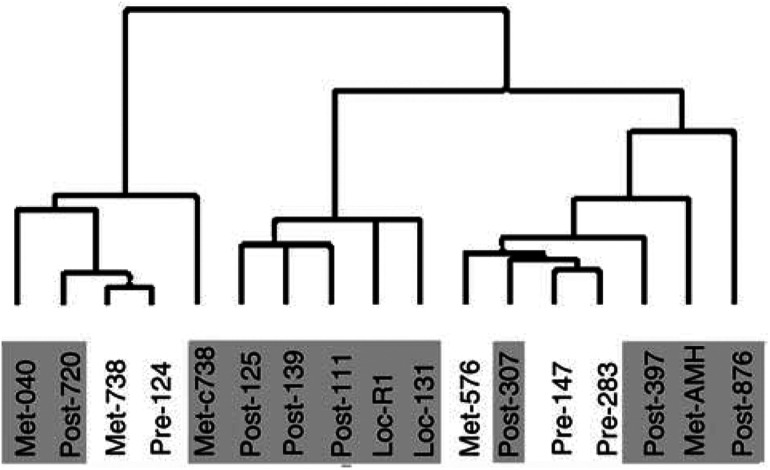
Sample clustering: clustering of the samples revealed little structure within the data. Full patient details are in Table 1, but briefly: Pre, primary site; Post, primary site/post chemotherapy; Met, metastasis; Loc, local recurrence. Patients with cytotoxic treatment immediately prior to resection are also shaded in red.

The data were then filtered to select approximately 1500 genes that varied most between samples (s.d. of log 2 data >0.5). In the subsequent hierarchical clustering of the samples (see Figure 2

), met-738 and the primary cell culture, we established from this lesion (Met-c738), clustered tightly together. This suggests that for array analysis, early passage primary cell culture, rather than laser capture microdissection (LCM) (Simone *et al*, 2000), could be useful to enrich for OS cells from a mixed tumour stromal microenvironment. This is fortunate as it is currently not technically possible to employ LCM on OS samples. However, it was difficult to see any further pattern to the data from this exploratory analysis.

**Figure 3:**
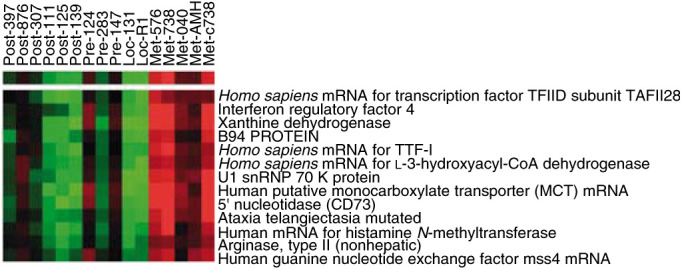
Metastatic heatmap: genes highly expressed in metastatic samples are shown (*P*<0.01). Note that both local recurrence samples show a pattern of expression similar to the primary samples. Post, postchemo primary; Pre, primary; Loc, local recurrence; Met, metastasis.

An analysis of genes differentially expressed between primary and metastatic samples is shown in Figure 3

. (*P*<0.01). Among the genes highly expressed in metastatic samples was the powerful antioxidant xanthine dehydrogenase often associated with cellular injury, and the genes associated with lymphocyte activation, CD73 and Interferon regulatory factor-4. The overexpression of genes in the metastatic samples appeared higher than in primary. Both local recurrence samples had a profile much more similar to primary than metastatic disease.

**Figure 4:**
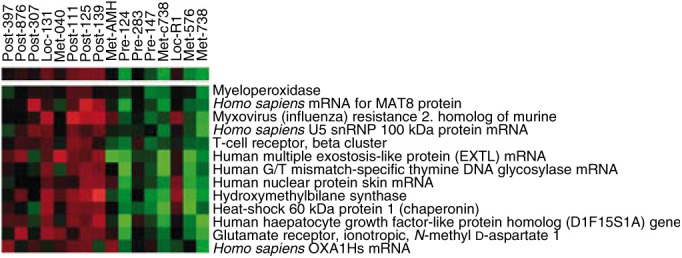
Chemotherapy heatmap: genes highly expressed in chemotherapy treated patients immediately prior to resection. All differences were significant at *P*<0.01. Post, postchemo primary; Pre, primary; Loc, local recurrence; Met, metastasis.

Likewise, we also examined differences between samples either cytotoxically treated immediately prior to resection Figure 4

. (*P*<0.01). Strikingly among the genes most highly expressed after cytotoxic treatment were the myeloperoxidase gene associated with intranuclear protection of DNA from damage by free radicals (Borregaard & Cowland, 1997) and the thymine DNA glycosylase that initiates repair of G/T and G/U mismatches after hydrolytic deamination of 5-methylcytosine (Lindahl, 1982; Lindahl *et al*, 1982) Hsp-60 was also a highly expressed factor that may correlate with apoptotic stress on the tumour cells.

We have demonstrated here that biopsies of bone tissue are suitable for extraction of sufficient good quality RNA for GEM analysis.
